# Nanoformulations of Herbal Extracts in Treatment of Neurodegenerative Disorders

**DOI:** 10.3389/fbioe.2020.00238

**Published:** 2020-04-07

**Authors:** Seyed Zachariah Moradi, Saeideh Momtaz, Zahra Bayrami, Mohammad Hosein Farzaei, Mohammad Abdollahi

**Affiliations:** ^1^Pharmaceutical Sciences Research Center, Health Institute, Kermanshah University of Medical Sciences, Kermanshah, Iran; ^2^Medical Biology Research Center, Kermanshah University of Medical Sciences, Kermanshah, Iran; ^3^Medicinal Plants Research Center, Institute of Medicinal Plants, ACECR, Karaj, Iran; ^4^Toxicology and Diseases Group, Pharmaceutical Sciences Research Center (PSRC), The Institute of Pharmaceutical Sciences (TIPS), Tehran University of Medical Sciences, Tehran, Iran; ^5^Department of Toxicology and Pharmacology, School of Pharmacy, Tehran University of Medical Sciences, Tehran, Iran

**Keywords:** herbal extracts, neurodegenerative disorders, nanoformulations, nanoparticles, Alzheimer’s disease, Parkinson’s disease

## Abstract

Nanotechnology is one of the methods that influenced human life in different ways and is a substantial approach that assists to overcome the multiple limitations of various diseases, particularly neurodegenerative disorders (NDs). Diverse nanostructures such as polymer nanoparticles, lipid nanoparticles, nanoliposomes, nano-micelles, and carbon nanotubes (CNTs); as well as different vehicle systems including poly lactic-co-glycolic acid, lactoferrin, and polybutylcyanoacrylate could significantly increase the effectiveness, reduce the side effects, enhance the stability, and improve the pharmacokinetics of many drugs. NDs belong to a group of annoying and debilitating diseases that involve millions of people worldwide. Previous studies revealed that several nanoformulations from a number of natural products such as curcumin (Cur), quercetin (QC), resveratrol (RSV), piperine (PIP), *Ginkgo biloba*, and *Nigella sativa* significantly improved the condition of patients diagnosed with NDs. Drug delivery to the central nervous system (CNS) has several limitations, in which the blood brain barrier (BBB) is the main drawback for treatment of NDs. This review discusses the effects of herbal-based nanoformulations, their advantages and disadvantages, to manage NDs. In summary, we conclude that herbal-based nano systems have promising proficiency in treatment of NDs, either alone or in combination with other drugs.

## Introduction

Neurodegenerative disorders (NDs) are defined as range of disruptions in function or structure of the nervous system or neurons. Such lasting progressive damages may cause disability in thinking, movement, cognition, and memory. Among various NDs, Alzheimer’s disease (AD) and other types of dementias; Parkinson’s disease (PD) and PD related disorders; Multiple sclerosis (MS); Huntington’s disease (HD); and Amyotrophic lateral sclerosis (ALS) are the most prevalent types. Genetic susceptibility, aging, lifestyle, nutrition, chemicals, specific viruses, and exposure to some environmental toxins (Przedborski et al., 2003; Hodjat et al., 2017) are supposed to be predominant risk factors of NDs. Nowadays, the global average of life expectancy increased, hence; the prevalence of age-related NDs is drastically rising. According to the World Health Organization (WHO) report on the top 10 causes of global death, the rate of mortality caused by dementia, and age related NDs has raised more than twice from 2000 to 2016, also dementia was the 5th cause of death in 2016 World Health Organization (2018). Thereby, it is predicted that mental and emotional defects will cause emotional, social, and financial burden on the healthcare system in the future World Health Organization (2006). Current treatments for NDs have considerable adverse effects, thus, there is still demand to seek new strategies with reduced harms (Durães et al., 2018). In this respect, natural products sound propitious, although, their penetration through the BBB is a major obstacle in their delivery to the nervous system (Dwivedi et al., 2019). In this manner, nanotechnology and more specifically, nanomedicine or pharmaceutical nanotechnology provide superior drug delivery systems for NDs management by means of improved monitoring, controlling, constructing, repairing, and diagnosis at a molecular level (Ochekpe et al., 2009; Maravajhala et al., 2012). Nanoformulations of these natural substrates are effective tactics to overcome such problems and can enhance their bioavailability (Ratheesh et al., 2017). This study reviews the recent efforts in the application of nanotechnology in formulation of natural drugs to improve NDs treatment.

## Neurodegenerative Disorders (NDs)

Previously, the CNS disorders were categorized as cognitive, motor, or combined impairments, mainly on the basis of patient’s symptoms. This classification faced several criticisms, since some symptoms were common between the groups, also because many symptoms did not fall into a specific category. Today, it has been proven that abnormalities in particular proteins such as amyloid precursor protein (APP), tau, and α-synuclein lead to NDs. Currently, the CNS disorders are being reclassified on the basis of the number of protein abnormalities (Rogan and Lippa, 2002).

### Alzheimer’s Disease (AD)

**FIGURE 1:**
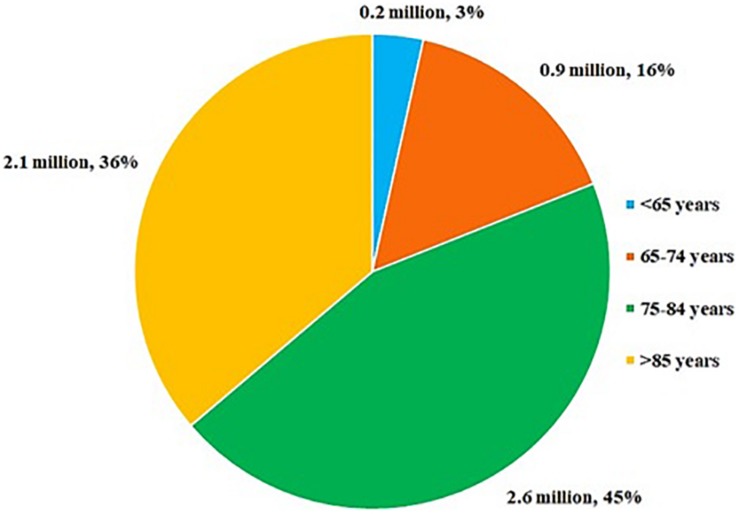
The average age of people with AD.

Alzheimer’s Disease and associated dementia have been listed as the 6th leading cause of death in the United States population (Heron, 2018). According to data published by the AD association in 2019, 5.8 million of American populations of all ages are living with AD. Age-based population is suffering from AD and related dementia shown in Figure 1Alzheimer’s and Dementia (2019). AD is a chronic incurable, progressive ND with a long pre-symptomatic period. Generally, AD is associated with cognitive impairments; behavioral, social and work related dysfunctions; and ultimately leads to death (Bateman et al., 2012). Aberrant accumulation of protein β-amyloid (β-amyloid plaques) outside the neurons, and abnormal accumulation of protein tau (tau tangles) inside the neurons are the major hallmarks of AD. The β-amyloid induces neuronal cell death through disrupting their communications at synapses, while tau tangles contribute to neuronal cell death by blocking the entry of nutrients and other essential molecules into the neurons. Gradual increase of β-amyloid plaques outside the neurons results in consequent spreading of tau tangles throughout neurons (De Paula et al., 2009; Penke et al., 2017; Momtaz et al., 2018).

Aberrant presence of toxic proteins activates the brain immune cells, as well as microglia cells. Microglial cells are specialized brain macrophages that are able to eliminate abnormal aggregated proteins, and debris from dead or dying neurons. Inflammation and atrophy are also associated with AD. Inflammation occurs when microglial cells are not capable of clearing all the things that are supposed to be eliminated, while neuronal loss leads to atrophy.

With time, β-amyloid plaques and tau tangles spread in other brain areas, which are not involved in cognitive functions (Price et al., 1991). Progressive damages to the brain cells initiate cognitive dysfunctions, and most importantly cause memory impairments (2019). Often, the word ‘dementia’ is used with AD, as the symptoms of dementia have coincided with the AD symptoms. Dementia refers to a group of symptoms related to cognitive, and memory decline. Furthermore, abnormal precipitation of protein α-synuclein inside the cortical neurons, and Lewy bodies results in dementia (Mckeith et al., 1996; Duda et al., 2000; Rogan and Lippa, 2002).

### Parkinson’s Disease (PD)

Parkinson’s Disease was reported in the early 18th by the physician Dr. James Parkinson as “shaking palsy”. PD is a chronic progressive ND that encompasses both motor- and non-motor dysfunctions, with deteriorating effects on mobility and muscle control (Demaagd and Philip, 2015). Current global burden of the disease has been more than doubled over the past 26 years, from 2.5 million patients in 1990 to 6.1 million patients in 2016 (Rocca, 2018). The main risk factors of PD include aging, environmental changes, chronic diseases, and social difficulties (Schrag et al., 2015). Continuous loss of dopaminergic neurons in the substantia nigra pars compacta results in loss of dopaminergic function in PD patients. In PD patients, progressive loss of dopamine in striatum leads to increased globus pallidus segment/reticulate portion of the substantia nigra circuit’s activity. This activity, consequently, leads to gamma aminobutyric acid (GABA) dysfunction, and inhibition of thalamus and motor activities (Beaulieu and Gainetdinov, 2011). Aberrant accumulation of Lewy bodies is also reported in PD patients (Braak et al., 2003; Del Tredici and Braak, 2012). Mutation in α-synuclein gene was shown to form insoluble fibrils in Lewy bodies (Yasuda and Mochizuki, 2010).

### Multiple Sclerosis (MS)

Multiple sclerosis is a chronic neurological disorder, leading to demyelination of the nerve cells in the brain and spinal cord. Such demyelination disrupts interneurons communication, persuading axonal loss in both white and gray matter of the brain and spinal cord, although the loss is more prominent in white matter (Kutzelnigg and Lassmann, 2014). MS is also categorized as an autoimmune disorder, in which T cells target the CNS self-antigen in genetically prone individuals. Initial lesions are mostly formed in focal areas of demyelinated white matter; these focal areas are called plaques. Pathological symptoms of MS vary with the locations of the plaques, but basically are associated with infiltration of immune T cells across the BBB (Polman et al., 2011). Demyelination and loss of trophic support in oligodendrocytes lead to axonal degeneration (Fünfschilling et al., 2012; Lee et al., 2012). Pathological aggregation of fibronectin was also observed in MS lesions. It was documented that the aggregation of this glycoprotein is likely associated with remyelination failure. In addition, tau protein, amyloid-β and amyloid precursor proteins, which are normally detected in AD and PD subjects, are also found in plaques and lesions of MS patients (Stoffels et al., 2013; David and Tayebi, 2014). MS subjects experience a series of relapsing-remitting courses, in which, there is an acute episode of neural impairments followed by normal baseline function. With time (after 10–15 years), relapses shift into inevitable progressive neurodegeneration, termed as secondary progressive MS (Scalfari et al., 2014). However, approximately 10–15% of patients directly enter the secondary neurodegenerative state, known as primary progressive MS. The length of the relapsing-remitting state shows considerable variations, however, the rate of neurodegeneration is highly consistent, irrespective of the disease course and severity (Friese et al., 2014).

### Amyotrophic Lateral Sclerosis (ALS)

Amyotrophic lateral sclerosis includes two major forms; sporadic and familial types. The sporadic form (prevalence of 90–95%) has no hereditary history, while the familial type (5–10%) has a genetically inherited component (Abhinav et al., 2007; Zarei et al., 2015). ALS is a heterogeneous neurological disorder; characterized by degeneration of both the upper and lower motor neurons (Logroscino et al., 2008, 2010). Besides cellular stress, it was suggested that the aggregation of intraneuronal proteins i.e., TAR DNA-binding protein 43 (TDP-43), superoxide dismutase (SOD1), and fused in sarcoma (FUS) disturb normal protein homeostasis, thereby inducing ALS. These proteins are well identified in pathological studies of patients with ALS and in animal models of the disease (Morgan and Orrell, 2016). Common symptoms of ALS include muscle tenderness, cramping, twitching, and muscle impairment (Goetz, 2000). Later in the advance stage of the disease, patients experience dysphagia (swallowing difficulty), dysarthria (speech difficulty), and dyspnea (difficulty in breathing) (Kori et al., 2016). Environmental pollutants and diet have also been investigated for their association with ALS (Morozova et al., 2008; Yu et al., 2014). Multidisciplinary approaches seem favorable for ALS management.

### Huntington’s Disease (HD)

Huntington’s disease is a monogenic autosomal dominant neurological disease. Due to its autosomal dominant inheritance pattern, progressiveness and the combination of motor/cognitive/and behavioral impairments, the disease condition is traumatic to patients and their relatives (Bates et al., 2015). Pathologically, HD is the result of an expanded trinucleotide repeat of CAG sequence in the gene *HTT5* on chromosome 4, encoding the abnormal pathogenic multifunctional protein named Huntingtin. Mutant protein holds an unusual polyglutamine sequence, corresponding to the expanded CAG repeat, which is known to be toxic in nature, and results in neuronal cell death or dysfunction. Neurons of the striatum region are particularly prone to this mutant protein; however, HD has been documented as a disorder of whole the brain and body. Abnormality of huntingtin protein leads to neuronal death through several mechanisms including direct effect of the mutant protein exon 1; and tendency of the mutant protein to form aggregates with direct effect on axonal transport, protein homeostasis, and mitochondrial function (Kay et al., 2014; Ross et al., 2014). Losses of the brain-derived neurotrophic factors, glutamate excitotoxicity, and toxic effects of repeat associated non-ATG translation are the other hypothesis involved in neural damage of HD (Bates et al., 2015).

## Clinical Strategies, Management, Challenges and Limitations Versus NDs

Recent couple of decades have witnessed exceptional researches that propelled our knowledge about NDs. Advances in genetic sciences enormously helped to target such diseases with novel technologies (Chen and Pan, 2014). A set of allopathic medicine such as dopaminergic medications for PD and related motor disorders (Mizuno, 2014); cholinesterase inhibitors for treating cognitive disorders (Doody et al., 2009); analgesic drugs for pain (Chaudhuri and Schapira, 2009); anti-inflammatory (Tizabi et al., 2014) and antipsychotic drugs for dementia and other behavioral dysfunctions (Desai and Grossberg, 2005) are used to stop the tremor and refractory movement disorders (Okun, 2014). Active and passive immunotherapies are new hopes for AD treatment, though, the adverse effects of these antibodies are the biggest concern of immune related drugs (Chen and Pan, 2014). Integrative medicine, including Western and traditional medicine, is also effective option in improving NDs (Pan and Zhou, 2014). For example, in PD patients, utilization of “Traditional Chinese Medicine” (TCM) and allopathic medicine helped to improve sleeping and associated non-motor disorders (Pan et al., 2011a, b). Ayurveda also has a history of PD treatment with lower side effects (Lloret et al., 2013). Moreover, TCM was shown to improve the symptoms of cognitive and behavioral impairments in AD subjects (Pan et al., 2014). Medicinal plants used in traditional Persian medicine have also shown notable advantages for NDs treatment (Farzaei et al., 2018b). In another study, a balanced based exercise regimen was shown to improve the postural stability in PD patients (Li et al., 2012). It is believed that combination of integrative medicine and modern science will gradually help to treat degenerative diseases. Despite considerable progressions in NDs management, certain limitations and challenges are yet to be addressed.

It is thought that NDs might be treatable by predicting pathological conditions prior to the onset of the disease, i.e., the biomarkers of human immunodeficiency viruses (HIV) (CD4 cell count or viral load). This idea is supported by the well-definition of pathological and clinical phenomena. In sporadic PD, the severity of the disease is measured by nonspecific markers. For instance, the majority of individuals with constipation will never get PD. Therefore, sorting of individuals to well defined risk groups is still a need that has to be fulfilled. Definition of decent outcomes and efficient biomarkers are necessary to show whether a participant is responding in preclinical and clinical trials.

Systemic delivery of drugs to the CNS is a significant challenge, mainly due to their poor access to the brain, extensive first-pass metabolism, limited half-life, and possible side effects when reaching non-target peripheral tissues (Tonda-Turo et al., 2018). The BBB and other barriers inside the CNS such as the meninges, blood cerebrospinal fluid barrier, choroid plexus within each brain ventricle, and circumventricular organs are the obstacles of drug delivery to the CNS. Hence, development of systemic delivery systems with increased efficacy is essential for the CNS pharmacotherapy.

### Medicinal Plants and Their Phytochemicals for NDs Treatment

Numerous studies tried to characterize phytochemicals with positive effects on the neural system from medicinal, and even dietary plants (Kim et al., 2014; Naoi et al., 2017, 2019). Beneficial effects of neuroprotective phytochemicals are mainly attributed to their antioxidant, and anti-inflammatory properties (Kim et al., 2010). The bioavailability of herbal bioactive components in body is a key point in their bioefficacy, and might be restricted by factors such as fast metabolism, trivial permeability, and the lack of stability within the CNS (Pandareesh et al., 2015). Herein, we list the main chemical groups involved in neuroprotection.

#### Polyphenols

**FIGURE 2:**
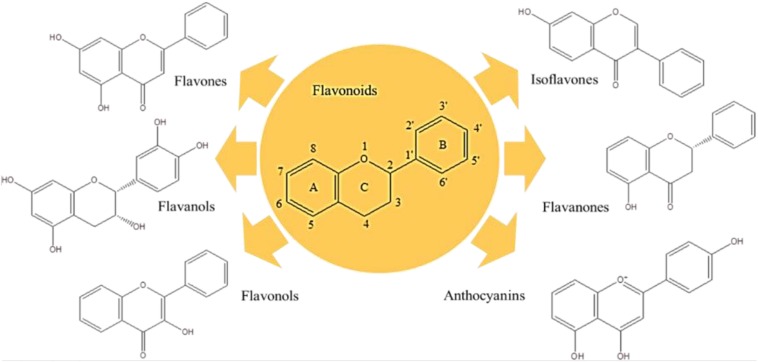
Flavonoids.

Polyphenols are the largest group of plant secondary metabolites, and their structures vary from hydroxyl groups attached to the aromatic ring in the simple phenols to highly complex polymeric compounds in tannins and lignins. Respecting their structures, polyphenols are strong antioxidant, and anti-inflammatory compounds with broad contribution to manage various diseases. To date, several clinical trials were proceeded to investigate the potency of polyphenols on different NDs (Goetz, 2000; Logroscino et al., 2010; Kori et al., 2016; Morgan and Orrell, 2016; Davatgaran-Taghipour et al., 2017). Flavonoids are the major bioactive group of polyphenols with more than 6000 members (Figure 2). Flavones (i.e., apigenin and luteolin) (Patil et al., 2014), flavanol (i.e., epigallocatechin-3-gallate-EGCG) (Singh et al., 2016), flavonols (i.e., QC and kaempferol) (Lagoa et al., 2009; Barreca et al., 2016), isoflavones (i.e., daidzein and genistein) (Qian et al., 2012; Aras et al., 2015), flavanones (i.e., naringenin and hesperetin) (Cirmi et al., 2016), and anthocyanins (i.e., cyanidin and delphinidin) (Strathearn et al., 2014) are the best known flavonoids with considerable medicinal and dietary values, particularly neuroprotective properties.

**FIGURE 3:**
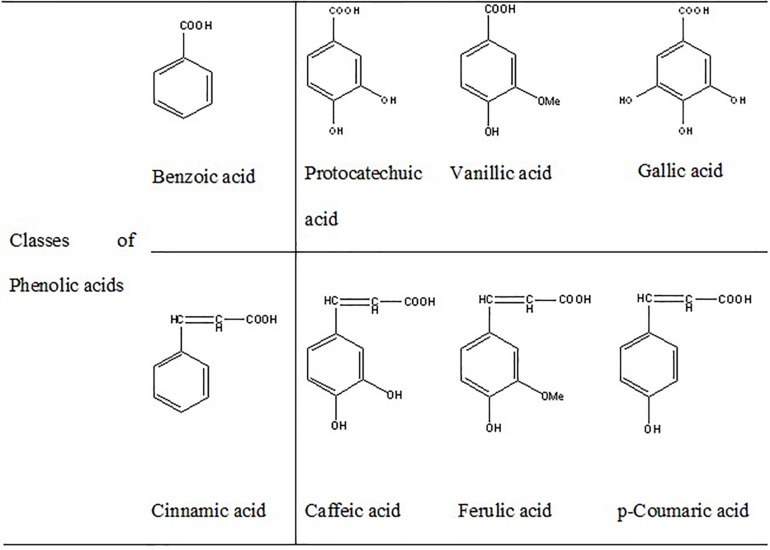
Phenolic acids.

Curcumin (diarylheptanoid) (Hu et al., 2015), RSV (stilbenoid) (Gomes et al., 2018), catalpol (iridoid glycoside) (Jiang B. et al., 2015), lycopene (carotenoid) (Prema et al., 2015), and smilagenin (saponin) (He et al., 2019) are some of the non-flavonoid polyphenols with significant neuroprotective effects. Phenolic acids containing cinnamic acid derivatives (i.e., *p*-coumaric acid, caffeic acid, ferulic acid), and the benzoic acid derivatives (i.e., gallic acid, vanillic acid, protocatechuic acid) have been reported to improve neurological dysfunctions through direct effect on neural, and glial cells (Nabavi et al., 2015; Szwajgier et al., 2017; Figure 3). Scavenging of reactive oxygen and nitrogen species, activation of redox-responsible transcription factors, regulation of gene expression, inhibition of β-amyloid generation and aggregation, as well as regulation of mitochondrial apoptosis system have been introduced as some of the mechanisms involved in the neuroprotective functions of polyphenols. Furthermore, it is suggested that polyphenols can bind to specific receptors on cell surface and trigger different antioxidant signaling pathways (Rehman et al., 2019).

#### Alkaloids

**FIGURE 4:**
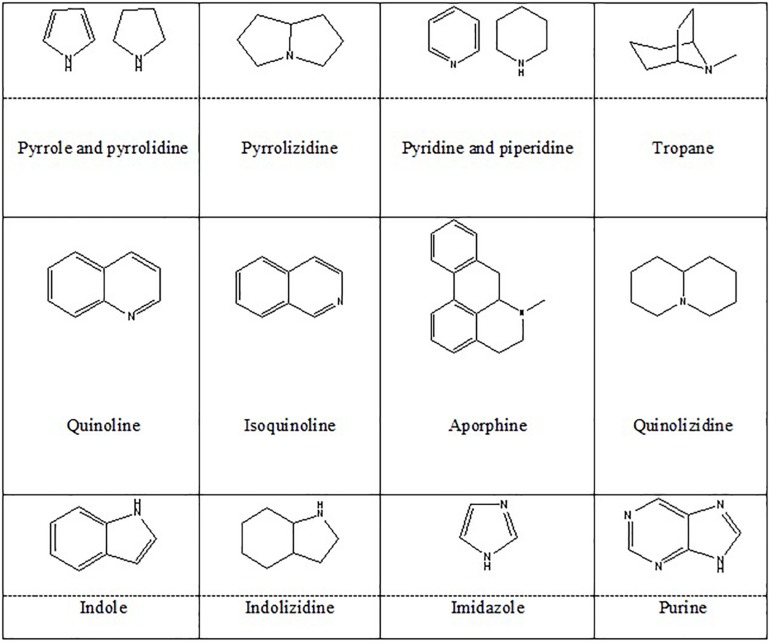
Heterocyclic alkaloids.

Alkaloids are organic natural compounds containing nitrogen in their structures. There are various classifications of alkaloids based on their chemical structures, biochemical precursors, and pharmacokinetics. Heterocyclic alkaloids (typical alkaloids) with nitrogen in their cyclic ring are more common. Berberine (*Berberis vulgaris*) (Jiang W. et al., 2015), montanine (*Rhodophiala bifida*) (Pagliosa et al., 2010), morphine (*Papaver somniferum*) (Wang et al., 2018), salsoline (*Salsola oppositifolia*), and galantamine (*Galanthus nivalis*) (Pagliosa et al., 2010) belong to isoquinoline alkaloids, and proven to have positive effects on NDs. In addition, PIP (a piperidine alkaloid from *Piper nigrum*) (Chonpathompikunlert et al., 2010), geissospermine (Vital et al., 2015) (an indole alkaloid from *Geissospermum vellosii*), nicotine (Quik et al., 2012) (a pyridine alkaloid from *Nicotiana tobaccum*), caffeine (a methylxanthine derivative from *Coffea arabica*) (Tellone et al., 2017), and harmine (an indole β-carboline from *Peganum harmala*) (Biradar et al., 2013) were also shown to possess neuroprotective effects. These species majorly belong to Amaryllidaceae, Papaveraceae, Solanaceae, and Ranunculaceae families of plant kingdom. Figure 4 represents the chemical structures of heterocyclic alkaloids. Alkaloids affect NDs through different mechanisms including modulation of neurotransmitter systems, inhibition of anti-amyloid and monoamine oxidase (MAO), inhibition of acetylcholinesterase and butyrylcholinesterase, inhibition of α-synuclein aggregation, and by anti-inflammatory and antioxidant activities. They also might act as dopaminergic and nicotine agonists or N-methyl-D-aspartate (NMDA) antagonist (Hussain et al., 2018).

#### Terpenoids

Terpenoids are unsaturated organic compounds composed of isoprene units. It was shown that *G. biloba* has positive effects on NDs and contains flavonoid glycosides, organic acids and terpenoids such as ginkgolides A, B, C and bilobalide (Shi et al., 2010). Thymoquinone (TQ), the major component of *Nigella sativa* is a monoterpene that has been suggested to be responsible for the neuroprotective property of the plant (Khazdair, 2015). Thymol is another neuroprotective monoterpene isolated from *Thymus vulgaris* (Deng et al., 2015). GABA mediated inhibition of synaptic transmission is the probable mechanism for the thymol neuroprotective effect (Marin et al., 2011).

### Advantages of Nanoparticles for Treatment of NDs

The presence of BBB is the main obstacle for NDs treatment strategies. So far, great efforts have been conducted to dispel this problem with help of various nano methods (Bhaskar et al., 2010). Among the drug delivery carriers that have been engineered, polymeric nanoparticles (PNPs) received particular interests, due to their high drug loading capacity, long circulation half-life, and high capacity to protect the drug against debasement, which offers broad surface handling possibilities for ligands to pass the BBB (Roney et al., 2005). Nowadays, there are claims that NP-based drug delivery systems effectively boost up the passage of drugs through the BBB and even raise the drug absorption in the brain. Biodegradability and reduced toxicity to peripheral organs are reported as the main advantages of nanomaterials for such therapeutic purposes (Caruso et al., 2011). Nanomaterials pass the BBB through non-invasive, and invasive mechanisms. In invasive manner, physical methods, the BBB is ruptured and nanomaterials are transported across the BBB through paracellular pathways such as intracerebroventricular or intracerebral injection, i.e., intranasal delivery strategy, receptor-mediated BBB crossing strategy, cell-mediated BBB crossing strategy, shuttle peptide-mediated BBB crossing strategy and cell-penetrating peptide (CPP). In contrast, non-invasive strategies preserve the basic structure of BBB during the drug delivery process and do not harm the BBB (Xie et al., 2019). Encapsulation inside the nanocarriers simplifies the drug entry into the brain through a non-invasive manner (Poovaiah et al., 2018). It is believed that nanocarriers can be engineered desirably without affecting or altering the medication’s properties.

In neural cells, in addition to the BBB, nanomaterials target free radicals production/activity and the oxidative related pathways (Win-Shwe and Fujimaki, 2011); regulate the inflammatory events (i.e., suppression and/or overexpression of pro- or inflammatory cytokines and chemokines); possess autophagy modulating (Zheng et al., 2016) and neuronal tissue regeneration effects (Re et al., 2012); also can suppress neural apoptosis or toxicity (Ali et al., 2017); and modulate the transcription, transduction, and intracellular signaling pathways (Kim et al., 2017). In AD subjects, nanostructures display high affinity for Aβ to reduce its toxic effects, while in PD case, nano-based approaches facilitate dopamine delivery and release to the brain (Re et al., 2012).

Treatment of NDs with NPs may have significant consequences such as proper biocompatibility and biodegradability, improvement of the drug pharmacokinetic and therapeutic efficacy, and reduction of the drug adverse effects (Ratheesh et al., 2017). It was reported that herbal Ginsenoside-NPs possessed neuroprotective effect, mainly through crossing the BBB (Aalinkeel et al., 2018). Poly Lactic-co-Glycolic Acid (PLGA)-functionalized QC (PLGA@QC)-NPs shown negligible cell toxicity, inhibited the Aβ_42_ fibrillation, and reduced the Aβ_42_-induced toxicity in human neuroblastoma SH-SY5Y cells *in vitro*. Novel Object Recognition and Morris Water Maze tests showed that PLGA@QC)-NPs treatment improved learning and memory impairments in AD mice (Sun et al., 2016). Bacoside (a loaded PLGA-NP) (Jose et al., 2014), Cholin-NPs (Li et al., 2011), Lectin-NPs conjugated with *Solanum tuberosum* lectin (Zhang C. et al., 2014), were also shown beneficial for AD management.

In summary, NPs loaded herbal extracts showed consequential effects on NDs by improving the drug biodegradability and biocompatibility, amelioration of the therapeutic efficacy, removing pharmacokinetics restrictions, reducing side effects, controlling the release, and by in site targeting. Furthermore, some of nanoparticle materials have the extra potential for enhancing the cure efficacy, such as decreasing ROS level, significant antioxidant properties, and even inhibiting the aggregation of Aβ. Although, nanocarriers are powerful tools for delivering specific compounds to the brain and can cross through the BBB in an easier manner, variant problems remained to be resolved. Smaller size may cause route dislocation, induce blood clots and hemolysis, thereby, creating platelet aggregation (Ramanathan et al., 2018; Niu et al., 2019). Imbalanced distribution of NPs in the brain may cause undeniable potential risks. Inorganic part of nanostructures such as gold, silica, iron, and cerium oxide particles make the metabolism of these NPs obscures.

Accumulation of these compounds in the brain can induce neurotoxicity through their impact on the mitochondrial activity and interference with autophagy, apoptosis, and neuronal inflammation (Niu et al., 2019).

## Herbal Medicines and Natural Compounds Nanoformulations

### Polymeric Nanoparticles (PNPs), Nanocapsules, and Nanospheres

Polymeric nanoparticles have high drug loading capacities, enabling the system to protect and support the incorporated drug against degradation. Therefore, there is an increasing chance of drug penetration and access to the brain. Due to their stable structures and unique features, they can evade macrophages, thus, facilitating the drug delivery to the CNS. Nanospheres are dense polymeric matrices that are prepared via micro-emulsion polymerization, while nanocapsules are developed by a thin polymeric envelope surrounding an oil-filled cavity (Modi et al., 2009, 2010; Ganesan et al., 2015).

### Polymeric Nanogels and Nanosuspensions

Nanogels are described as highly crosslinked nano-sized hydrogel systems that are either non-ionic- or ionic- monomers or copolymerized. The size of the nanogels varies from 20 to 200 nanometers. This system has a 40–60% capacity for drug loading. Previous studies suggested that nanogel structures could enhance the brain uptake and decrease the liver and spleen uptake of oligonucleotides. Drug loaded nanosuspensions are crystalline drug particles that have been stabilized by mixtures of lipids or nonionic surfactants. Nanosuspensions have significant advantages such as their simplicity to use, and their notable capacity for drug loading and delivery (Modi et al., 2010; Jain et al., 2019).

### Carbon Nanotubes (CNTs) and Nanofibers

Inorganic nano-drug delivery systems such as mesoporous silica nanoparticles, CNTs, layered double hydroxides, superparamagnetic iron oxide nanoparticles, and calcium phosphate nanoparticles emerged therapeutic applications in various diseases, particularly NDs. Inorganic nano-carbon systems are able to pass prolonged systemic circulation; while enhancing the drug accumulation, permeability, retention effect, stability, and availability to desire sites. In addition, these nanostructures could modulate the drug release, and facilitate drug imaging, and monitoring its function. Besides, being flexible to various stimuli (i.e., temperature, pH, chemicals, pressure, and magnetic and electric fields) makes the CNTs a great catch for nanopharmacology (Naz et al., 2019).

Utilization of carbon-based nanostructures, like CNTs, is one of the most noteworthy strategies for neurological applications. CNTs are allotropes of carbon with a cylindrical nanostructure. CNTs are being extensively explored to ameliorate their electrical stimulation. One of the effective options for treatment of different psychiatric and neurologic disorders, especially PD, is Deep Brain Stimulation. In some cases, the immune system reacts to the presence of these stimulating electrodes, arising problems for the utilization of such fibers. Fabrication of nanofibers is safer than CNTs, and the risk of air pollution is lower. Interestingly, nanofibers are used to design and produce neural prosthetics. Other nano methods may not be able to accomplish the same applications in comparison with the electrospun nanofibers (Modi et al., 2009, 2010; Ganesan et al., 2015). Considering their electronic properties, structural attributes, and suitable biological effects on the growth and viability of cells; CNTs can be applied as scaffolds alone or blending with other biodegradable biomaterials to promote neuro-engineering, for purposes like neuroprotection, neuronal differentiation, regeneration, interface, and stimulation (Xiang et al., 2019).

### Polymeric Nanomicelles

Polymeric micelles are among the most promising delivery systems in nanomedicine. This system has a core-shell structure with a lipophilic core, and a shell composed of hydrophilic polymer blocks. The main advantage of this system is the presence of hydrophobic active ingredients. The size of the polymeric micelles varies from 10 to 100 nanometers (Modi et al., 2009, 2010; Ganesan et al., 2015).

### Polymeric Nanoliposomes

**FIGURE 5:**
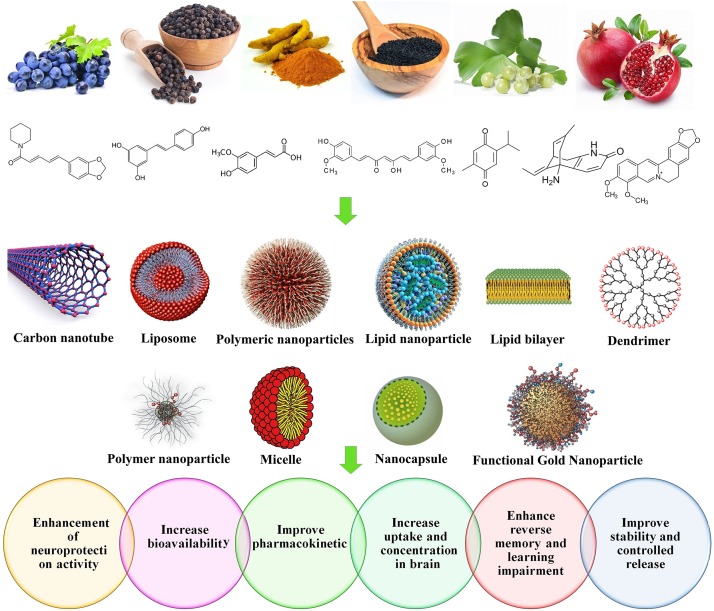
Nanoformulations used to improve the effectiveness of natural compounds.

Nanoliposomes are phospholipids with two hydrophobic tails and a hydrophilic head. Their sizes differ from 30 nanometers to few microns. A significant amount of drugs can be incorporated into the lipid bilayers or within the liposome aqueous compartments. Nanoliposomes with modified surfaces can decrease the drug opsonization in plasma, reduce the liver chance to eliminate such as liposomes, and increase their systemic circulation times. *In vitro* studies proven their efficiency for targeted CNS drug delivery, and confirmed their remarkable abilities to transfer a wide range of drugs from the BBB (Modi et al., 2009, 2010; Ganesan et al., 2015). Figure 5 represents the nanoformulations that are used to improve the effectiveness of natural compounds.

### Exosomes: New Promising Nanocarriers

Exosomes are lipid bilayer enclosed extracellular vesicles with nanometer-size ranging from 30 nm to 150 nm and are constructed in the endosomal compartment of the majority of eukaryotic cells such as B and T cells, dendritic cells, and macrophages. Exosomes have several special features that make them extraordinary and distinct from other nanocarriers. High biocompatibility; nanoscopic size; ability to communicate between cells, both systemically and locally; light immunogenicity; having remarkable potential to prevail over biological barriers; considerable potential for tissue targeting; encapsulation and carrying of various categories of unstable therapeutic molecules such as lipids, hormones, proteins, and genes; have enumerated the exosomes as suitable promising transporters for improving the drug delivery for treatment of multiple disorders such as NDs, cardiomyopathies and cancers (Aryani and Denecke, 2016; Sarko and Mckinney, 2017; Niu et al., 2019; Zhang et al., 2019).

Considering NDs, exosomes can play dual roles by either assisting in spreading the risky proteins such as prions, α-synuclein and tau, thereby, accelerating the progression of the disease; or due to their function in transporting cellular entities through the BBB, they can facilitate drug delivery to the brain and reduce the probability of NDs. In addition to the surface localizations of specific proteins, the presence of specific molecules that are known as risk factors for NDs and protecting their contents from degradation, make exosomes proper diagnostic candidates for NDs (Jan et al., 2017).

## Nanoformulation of Natural Products for NDs Treatment

### Curcumin (Cur)

Curcumin is one of the most popular and important natural polyphenols derived from *Curcuma longa* L. Cur has a distinctive chemical structure, making it susceptible to significant effects. Cur affects many biological and pharmacological targets, such as transcription factors, growth factors, genes and cytokines (Bengmark and Nutrition, 2006; Shao-Ling et al., 2009; Guo et al., 2017; Taghipour et al., 2019). Cur can modulate the inflammation process through suppression of several pro- and/or anti-inflammatory mediators such as tumor necrosis factor alpha (TNF-α), cyclooxygenase-2 (COX-2), and interleukin 8 (IL-8). Moreover, it has been reported that Cur is effective on Aβ and other protein aggregation, making it favorable to improve NDs, primarily AD (Zhang et al., 2010; Hajialyani et al., 2018; Del Prado-Audelo et al., 2019). Despite its impressive therapeutic properties, Cur showed inappropriate pharmacokinetics, in terms of limited absorption, negligible bioavailability, and fast elimination from the body. To overcome such defects, various NPs, nanocapsules, nanomicelles and nanoliposomes were developed to improve the pharmacokinetics and the bioavailability of Cur (Bollimpelli et al., 2016).

#### *In vitro* Studies

Curcumin loaded lactoferrin NPs were developed to protect SK-N-SH dopaminergic cells from rotenone-induced neurotoxicity, a model that mimics symptoms similar to PD. Besides sustained retention, the intracellular uptake, and the concentration of Cur increased, thereby, enhancing its neuroprotective effects (Bollimpelli et al., 2016). *In vitro*, a lipid- polyethyleneglycol-polylactide (PEG)-Cur derivative significantly reduced the Aβ aggregation. It was reported that Cur-derivative liposomes and Cur-derivative anti-transferrin antibody liposomes improved the brain penetration of the drug in *post-mortem* samples of AD patients (Mourtas et al., 2014). *In vitro*, nanoliposomes of Cur or Cur derivatives were able to control or decrease the Aβ oligomers or the fibril formation (Taylor et al., 2011). A double-functionalized nanoliposomes of a Cur derivative in modified HIV Trans-activating Transcriptional Activator (TAT) peptide (TATCur-NL) could cross the BBB *in vitro* and illustrated high affinity for Aβ peptides (Sancini et al., 2013). In another *in vitro* study, apolipoprotein E3 mediated poly(butyl) cyanoacrylate NPs containing Cur (ApoE3-C-PBCA), enhanced the photostability and the cellular uptake of Cur due to a sustained drug release (Mulik et al., 2010). PLGA coated-Cur NPs conjugated with Tet-1 peptide found suitable for treating AD, due to their inhibitory effects on Aβ formation and the consequent prevention of oxidation and production of free radicals (Mathew et al., 2012). In addition, Cur-decorated nanoliposomes displayed extremely high affinity for Aβ_1–42_ fibrils (Mourtas et al., 2011). Cur conjugated to a zwitterionic polymer (carboxybetaine methacrylate)-NPs, more effectively inhibited the fibrillation of Aβ_42_ fibrils than free Cur (Zhao et al., 2018). In another study, Cur-PLGA-NPs induced neurogenesis in neural stem cells through up-regulation of the expression of genes involved in neuronal differentiation and cell proliferation (Tiwari et al., 2013).

#### *In vivo* Interventions

Curcumin encapsulated solid lipid nanoparticles (CSLNs) improved 3-nitropropionic acid (3-NP)-induced HD in rats. CSLNs treated animals showed significant enhancement of the antioxidant enzyme’s activities (i.e., SOD and glutathione), while there was a significant decrease in mitochondrial swelling, ROS, protein carbonyls, and lipid peroxidation (Sandhir et al., 2014). Cur-selenium-PLGA nanospheres were shown more efficient in AD mice in comparison with only treated selenium-NPs animals (Huo et al., 2019). In another study, a dual drug-loaded lipid-based nanoformulation (Cur and PIP) found effective on PD. This effect was linked to the suppression of α-synuclein aggregation, enhancement of Cur bioavailability, alleviation of oxidative stress, more efficient removal of defective proteins, and acceleration of anti-apoptotic events compared with non-formulated drugs (Kundu et al., 2016). In another study, Cur NPs designed as vectors. These Cur-vectors showed considerable affinities toward Aβ_1–42_ fibrils and exhibited proper stability/integrity for *in vivo* applications (Mourtas et al., 2011). In mouse Tg2576 AD model, encapsulated PEG-PLA-Cur improved memory cue compared with control samples, also working memory was more improved in PEG-PLA-Cur treated mice than the ordinary Cur treated group (Cheng et al., 2013). In addition, solid lipid nanoparticles of Cur (Cur-SLNs) exhibited neuroprotective effects in aluminum-induced behavioral, biochemical and histopathological alterations in the mice brain (Kakkar and Kaur, 2011). Cur-loaded lipid-core nanocapsules (Cur-LNC) improved neuroinflammation, behavioral impairments and reduced the hyperphosphorylation of tau and Aβ in AD subjects, compared with free Cur treated animals (Hoppe et al., 2013). The safety and efficacy of a micelle nano-Cur system was reported in patients with ALS. The system increased the probability of survival in patients with ALS as an additional treatment, particularly in those with bulbar symptoms (Ahmadi et al., 2018). Encapsulated Cur in chitosan-alginate-sodium tripolyphosphate nanoparticles (CS-ALGSTPP NPs) augmented the bioavailability and the half-life of Cur in animal model of MS. Cur-loaded NPs reduced the inflammation, glial activation, and the extent of demyelination areas (Naeimi et al., 2018). In animal model of MS, dendrosome NPs of Cur ameliorated the score of the disease and demyelination, whereas the remyelination was improved, resulting in reduced inflammation and oxidative stress (Mohajeri et al., 2015). Another liposomal mucoadhesive drug delivery system has been shown effective on Cur delivery via nasal route. The system enhanced the drug bioavailability, distribution and stability, also controlled the release characteristics compared with the drug solution alone (Samudre et al., 2015).

In another study, Cur loaded to a low-density lipoprotein (LDL)-mimic nanostructured lipid carrier (Lf-mNLC) that was amended with lactoferrin. Administration of Lf-mNLC to AD animals enhanced the concentration of Cur in the brain and significantly increased its bioavailability, indicating that Lf-mNLC remarkably controlled the AD progression and symptoms (Meng et al., 2015). It was reported that a type of nano-Cur showed beneficial effects in restoring the expression patterns of dysregulated miRNAs in MS patients (Dolati et al., 2018a). In MS subjects, this system repressed the expression levels of T-helper 17 (Th17) cells, IL-17, and Retinoic acid-related orphan receptor gamma t (RORγt), demonstrating that this nano-Cur structure can prompt the regulation of dysregulated Th17 cells in MS patients (Dolati et al., 2018a). In another study, the effect of a nano-Cur system on regulatory T-cells frequency and function were investigated in 50 patients with relapsing-remitting MS. The system diminished the expression of forkhead box P3 (FOXP3) and the levels of IL-10, and transforming growth factor beta (TGF-β). In addition, the proportions of peripheral Treg cells were frequency declined, proposing that such nano-system is a competent agent to restore the frequency and function of Treg cells, which play an important role in MS patients (Dolati et al., 2019). Similarly, the very same nano-Cur decreased the expression levels of inflammatory miRNAs, signal transducer and the activator of transcription 1 (STAT1), nuclear factor-κB (NF-κB), and activator protein 1 (AP-1), while enhancing the expression of STAT5 mRNA (Dolati et al., 2018b). *In vivo* aggregation of Aβ_1__–__16_ was diminished using a gold nanoparticle– polyvinylpyrrolidone–Cur conjugate (Brahmkhatri et al., 2018). A Cur-loaded polysorbate 80 (PS80)-modified cerasome NPs caused longer circulation lifetime, and significantly improved the pharmacokinetic properties of the drug than free Cur in PD model (Nisi Zhang et al., 2018). A summary of Cur nanoformulations is provided in Table 1.

**TABLE 1:** Summary of Cur nanoformulations and their beneficial effects.

Nano vehicle/method	Disease	Results	References
Lactoferrin nanoparticles	Brain targeting, neuroprotection activity	Increase of intracellular drug uptake and higher neuroprotection properties	Bollimpelli et al., 2016
Multifunctional liposomes	AD	Decrease of Aβ_1–42_ aggregation and improve of pharmacokinetics of Cur	Mourtas et al., 2014
Liposomes	AD	Decrease of Aβ fibrils formation	Taylor et al., 2011
TAT	AD	High affinity for Aβ peptide and increase of Cur bioavailability	Sancini et al., 2013
Apolipoprotein E3 mediated poly(butyl) cyanoacrylate	AD	Increase of Cur bioavailability and photostability	Mulik et al., 2010
Se-PLGA nanospheres	AD	Reduction of amyloid-β aggregation	Huo et al., 2019
PLGA-based NPs	AD	Increase of Cur bioavailability and efficacy	Mathew et al., 2012
Nanoliposomes	AD	High affinity for Aβ_1–42_ fibrils	Mourtas et al., 2011
Poly(carboxybetaine methacrylate) (pCB)	AD	Improve of pharmacokinetics of Cur. Inhibition of Aβ_42_ fibrillation	Zhao et al., 2018
Lipid-based NPs	PD	Increase of bioavailability and reduce the aggregation of alpha-synuclein fibrils	Kundu et al., 2016
Liposomes	AD	Increase of affinity for Aβ_1–42_ fibrils and improve of pharmacokinetics quality	Mourtas et al., 2011
Polyethyleneglycol-polylactide (PEG-PLA)	AD	Increase of Cur bioavailability	Cheng et al., 2013
CSLNs	HD	Reduction of mitochondrial swelling, ROS, lipid peroxidation and protein carbonyls	Sandhir et al., 2014
SLNs	AD	Recuperation the noxious neurodegenerative effects of aluminum chloride	Kakkar and Kaur, 2011
Lipid-core nanocapsules	AD	Increase of Cur bioavailability	Hoppe et al., 2013
Micelle	ALS	Improve of probability of survival	Ahmadi et al., 2018
CS-ALGSTPP NPs	MS	Increase of Cur bioavailability, circulation and durability, inhibition of demyelination Preserve myelinated axons through amelioration	Naeimi et al., 2018
Dendrosome nanoparticles	MS	Improve of remyelination, decrease of inflammation and oxidative stress	Mohajeri et al., 2015
Mucoadhesive Liposome	AD	Good stability, controlled release, higher drug distribution and bioavailability	Samudre et al., 2015
Lactoferrin	AD	Improve the bioavailability and increase of brain penetration	Meng et al., 2015
Nano-micelle	MS	Restore the expression pattern of dysregulated miRNAs	Dolati et al., 2018a
Nano-micelle	MS	Decrease in Th17	Dolati et al., 2018a
Nano-micelle	MS	Suppression of Treg cell, IL-10, TGF-β, and FoxP3 expression	Dolati et al., 2019
Nano-micelle	MS	Suppression of inflammatory miRNAs, STAT1, NF-κB, and AP-1; increase the expression of STAT5 mRNA.	Dolati et al., 2018b
Gold nanoparticle–PVP	AD	Inhibit the Aβ_1–16_ aggregation and dissolve the formed aggregates	Brahmkhatri et al., 2018
PS80 modified cerasome	PD	Improve of pharmacokinetic profile	Nisi Zhang et al., 2018
PLGA	AD	Improve neuronal cell proliferation and differentiation, recuperation memory and learning disability	Tiwari et al., 2013

### Quercetin (QC)

Quercetin is a bioflavonoid found in diverse fruits, vegetables, and a number of herbal origin oils with well-known neuroprotective, and anti-inflammatory effects. Besides, QC has considerable potency to scavenge ROS. Despite its beneficial effects, poor solubility and low bioavailability hindered its clinical applications. Accordingly, to control such limitations, alternative QC formulations such as nanocapsules, nanogels, liposomes, nanosuspensions, and microsphere have been recommended, in which QC-nanocapsulation was shown to be the most proper form (Chakraborty et al., 2012; Nathiya et al., 2014; Ghaffari et al., 2018). In a study, QC-loaded nano lipidic carriers (NLCs) improved the QC bioavailability and delivery to the brain, while enhanced its antioxidant activity (Kumar et al., 2016).

In PD-like rats, the bioavailability and the efficacy of QC nanocrystals were greater than QC alone. A significant enhancement of the antioxidant enzyme activities and total glutathione level, as well as decline in malondialdehyde level were evident in hippocampal area (Ghaffari et al., 2018). Nanoencapsulated QC improved the ischemia reperfusion induced neuronal damage *in vivo*, probably in association with enhanced neuronal count and elevated antioxidant activity (Ghosh et al., 2013). *In vitro*, QC-SLNs significantly ameliorated aluminum induced neurotoxicity. In addition, this system caused meaningful improvement in behavioral and memory retention in animal models of dementia and AD (Dhawan et al., 2011). An ApoE-QC-RA-PA liposome structure (QC- and RA-loaded liposome with conjugated phosphatidic acid and grafted apolipoprotein E) was shown to cross the BBB and to recover the neurotoxicity of Aβ_1–42_ in AD model. *In vivo* AD model, the same system reduced the lipid peroxidation level, acetylcholinesterase activity and the formation of Aβ plaques (Kuo et al., 2018). Nasal administration of QC liposomes decreased the degeneration and destruction of cholinergic neurons in the hippocampus of AD animal model through reduction of oxidative stress (Phachonpai et al., 2010). In another study, a nanoformulation of QC (nano encapsulated QC) was designed and examined on neuronal model of oxidative stress injury. The neuroprotective activity of encapsulated QC was more explicit in comparison with free QC treated animals (Aluani et al., 2017).

The mitochondria delivery of QC increased by QC loaded in PLGA nanocapsules containing dodecyl triphenylphosphonium bromide (TPP+) as one of the matrix portions (N1QC) structure in the cerebral ischemia reperfusion induced model. N1QC showed higher brain uptake, and significant bioavailability and mitochondrial localization after cerebral ischemia-reperfusion (Ghosh et al., 2017).

### Resveratrol (RSV)

Resveratrol (3,5,4′-trihydroxy-stilbene) is a natural phytoalexin polyphenolic agent from the stilbene-class of compounds. Rapid metabolism, poor water solubility and low bioavailability are the main drawbacks of RSV (Farzaei et al., 2018a; Min et al., 2018). In PD mouse model, RSV loaded on PS80-coated poly(lactide) NPs increased the neuroprotective properties of the drug against 1-methyl-4-phenyl-1,2,3,6-tetrahydropyridine (MPTP)-induced behavioral and neurochemical variation (Da Rocha Lindner et al., 2015). An optimized RSV-loaded lipid-core NPs (RSV-LNC) modulated the Aβ-triggered neuroinflammation *in vitro* (Frozza et al., 2013a). Furthermore, RSV-LNC restored the destructive effects of Aβ_1–42_ in rats (Frozza et al., 2013b). In another study, RSV loaded mesoporous nano-selenium (MSe-Res/Fc-β-CD/Bor) delivery system inhibited the Aβ aggregation, decreased oxidative stress, and improved memory impairments (Sun et al., 2019). Similarly, RSV-loaded polymeric micelles inhibited the Aβ-induced damages via reducing oxidative stress and apoptosis *in vitro* (Lu et al., 2009). Vitamin E loaded RSV nanoemulsion showed notable positive effects in PD animal model and a higher concentration of RSV was detected in the brain in comparison with free drug treated group (Pangeni et al., 2014). In another study, RSV-loaded SLNs functionalized with apolipoprotein E, enhanced the bioavailability, concentration and the penetration of the drug in the brain (Neves et al., 2016). Chitosan-coated PLGA NPs of RSV reduced the level of inflammatory cytokines, elevated the IL-10 level, improved neuroprotection and enhanced the functional recuperation, following spinal cord damage in rats (Wang et al., 2019).

### Piperine (PIP)

Piperine (1-piperoylpiperidine) is a pungent alkaloid existing in the fruits of piper species. Bulk of evidence confirmed the effectiveness of PIP on the CNS, which is mainly implicated with the special consequences of PIP on acetylcholine. The log *P*-value of PIP is 2.25, making this compound very lipophilic, with slight aqueous solubility. In addition, PIP has insufficient oral bioavailability (Elnaggar et al., 2015b; Etman et al., 2018). A research group designed a Tween-modified monoolein cubosomes (T-cubs) loaded by PIP. In AD model, PIP-loaded cubs demonstrated higher efficacy over free drug and were able to restore the cognitive function in studied animals (Elnaggar et al., 2015b). Likewise, PIP microemulsion displayed higher efficacy, better therapeutic outcomes and increased the delivery of PIP to the brain compared with free drug in AD subjects (Etman et al., 2018). Intranasal PIP-loaded chitosan nanoparticles showed more efficacy with lower piperine dosage than piperine alone in AD model (Elnaggar et al., 2015a). In another study, nanoformulations of EGCG alone or in combination with PIP, improved cognitive behavior and reduced the brain acetylcholinesterase level in scopolamine-induced amnesia animals (Dahiya et al., 2018). PIP-SLNs formulated via emulsification solvent diffusion method coated with PS80, diminished the SOD1 level and immobility, while increasing the acetylcholinesterase level. Furthermore, reduced plaques and tangles in histopathological evaluation was evident (Yusuf et al., 2013).

### Gallic Acid (GA) and Epigallocatechin-3-Gallate (EGCG)

Gallic acid is a natural phenolic antioxidant synthesized from 3-dehydroshikimate. GA is suggested to play a protective role against α-synuclein and β-amyloid aggregations, both *in vitro* and *in vivo* (Nagpal et al., 2013; Jayamani and Shanmugam, 2014; Mohammad-Beigi et al., 2016). EGCG, is an ester of GA and epigallocatechin, the predominant catechin in tea. Regarding their antioxidant potencies, these compounds may have beneficial effects on NDs by their interactions with important proteins like α-synuclein, Aβ, huntingtin and transthyretin. GA loaded onto polyethyleneimine-coated human serum albumin nanoparticles (PEI-HSA-GA NPs) was shown to inhibit α-synuclein fibrillation in a PD model (Mohammad-Beigi et al., 2016). In another study, GA-loaded chitosan nanoparticles (GANP) recuperated scopolamine-induced amnesia *in vivo*. This effect was mainly ascribed to GA cholinergic function and its antioxidant properties. Furthermore, GANP coated with Tween 80 (cGANP) reinforced the above-mentioned effects of GA (Nagpal et al., 2013). Nanolipidic EGCG improved the bioavailability and neuronal α-secretase activity of EGCG in AD and HIV-associated dementia mouse models (Smith et al., 2010). To decrease the cytotoxicity of EGCG at high doses, EGCG was coupled on to the surface of selenium NPs coated with Tet-1 peptide (Tet-1-EGCG@Se). This system inhibited the Aβ fibrillation and disaggregated the Aβ fibrils into the non-toxic compounds (Zhang J. et al., 2014).

### Ferulic Acid (FA)

Ferulic acid is a cinnamic acid derivative with strong antioxidant activity. The compound can reduce the Aβ fibrils formation, thus may affect AD (Picone et al., 2009; Mhillaj et al., 2018). The pharmacokinetics and bioavailability of FA were found to be insufficient, thereby, restricting its therapeutic applications (Trombino et al., 2013). It was shown that the pharmacokinetic and delivery profile of FA was enhanced by SLNs system. In rat brain microsomes, FA-SLNs recovered cell viability and mitochondrial membrane potential, inhibited Aβ-induced cell death, decreased ROS production, and reduced the activation of the apoptosis pathway. Two formulations of FA, SLNs-SA-FA (stearic acid) and SLNs-SF-FA (stearyl ferulate) based solid lipid NPs, were developed with more lipophilic properties than free FA. In addition to bioavailability, the antioxidant effect of FA in the rat brain was increased (Trombino et al., 2013). FA was also entrapped into multiple SLNs, and nanostructured lipid carriers (NLCs). *In vitro* AD model, ROS production decreased in human neuroblastoma LAN−5 cells treated with FA-loaded SLN, representative of higher protective activity of FA-nanoformulation in neurons than free FA (Bondi et al., 2009). In another investigation, FA-NLCs improved the pharmacological properties of FA via activation of phosphoinositide 3-kinases (PI3Ks) pathway in ischemic neural injuries model (Hassanzadeh et al., 2018).

### Plant-Mediated Nano Systems

Trimethylated chitosan-conjugated PLGA NPs (TMC/PLGA–NP) loaded with 6-coumarin and coenzyme Q10 improved memory impairment and reduced the senile plaques in transgenic mice. Moreover, it was shown that 6-coumarin loaded TMC/PLGA–NPs were highly accumulated in different parts of the brain in CD-1 mice, following intravenous injection (Wang et al., 2010). Intranasal delivery of Huperzine A (HupA)-loaded PLGA NPs (their surfaces were modified with lactoferrin-conjugated N-trimethylated chitosan) showed that Lf-TMC NPs facilitated the distribution of HupA in the brain. Furthermore, cellular uptake experiments demonstrated that accumulation of Lf-TMC NPs was higher than nontargeted analogs in SH-SY5Y and 16HBE cells. HupA-PLGA-NPs improved the bioavailability and targeting ability of the drug (Meng et al., 2018). In AD rat model, berberine (BRB)-loaded multiwalled carbon nanotubes (MWCNTs) coated with phospholipid and polysorbate, remanded the memory impairment and reduced the β-amyloid induced-AD compared with its free form (Lohan et al., 2017). In AD animals, hesperetin nanocrystal retrieved memory consolidation by upregulation of the antioxidant enzymes and glutathione levels (Kheradmand et al., 2018). PEG-based nanospheres encapsulated with vitamin E increased the antioxidant efficacy of vitamin E against Aβ-induced ROS (Shea et al., 2005). Retinoic acid-loaded polymeric NPs exhibited neuroprotective effects on dopaminergic neurons in mouse model of PD. This formulation significantly reduced dopaminergic neuron loss in the substantia nigra, while the expressions of transcription factors Pitx3 and Nurr1 were increased (Esteves et al., 2015). In Aβ_25–35_ induced oxidative stress in rat hippocampal region, chrysin loaded SLNs showed potent free radical scavenging effect, decreased neuronal damage and improved oral bioavailability; also slight memory retention in behavioral tasks was observed (Vedagiri and Thangarajan, 2016).

Sialic acid (SA)-modified selenium NPs coated with peptide-B6 (B6-SA-SeNPs) enhanced the penetration of the drug across the BBB, effectively disaggregated the Aβ fibrils and inhibited its aggregation (Yin et al., 2015). Cysteine-modified SeNPs (D/LSeNPs) diminished ROS level and prevented metal-induced Aβ aggregation. Furthermore, D/SeNPs showed a higher inhibitory effect on fibrils formation than L/SeNPs *in vitro* (Zhou et al., 2015). In the same way, (PLGA)-encapsulated nattokinase conjugated with Tet1 peptide exhibited antifibrinolytic activity and downregulated the amyloid aggregation (Lohan et al., 2017) (Table 2).

**TABLE 2:** Natural-based nanoformulations and their implications for NDs.

Component	Nano vehicle/method	Disease model	Results	References
Coenzyme Q10 6-coumarin	Trimethylated chitosan-conjugated PLGA nanoparticle	AD	↓ Senile plaques ↓ Memory impairment, ↑ bioavailability	Wang et al., 2010
HupA	lactoferrin-conjugated N-trimethylated chitosan nanoparticles (Lf-TMC NPs)	AD	Appropriate sustained-release, ↑ bioavailability, ↑ targeting ability	Meng et al., 2018
Berberine	MWCNTs coated with phospholipid and polysorbate	AD	Remanded the memory impairment and quelled AChEI activity	Lohan et al., 2017
Hesperetin	Nanocrystal	AD	Improve derecognition of memory consolidation ↑ Activity of antioxidant enzymes	Kheradmand et al., 2018
Vitamin E	PEG-based nanospheres	AD	↑ Antioxidant efficacy of vitamin E	Shea et al., 2005
Retinoic acid (RA)	PNPs	PD	Significant neuroprotective effect on dopaminergic neurons	Esteves et al., 2015
Chrysin	SLNs	AD	↑ Oral bioavailability, ↑free radical scavenging, ↓ neuronal damage	Vedagiri and Thangarajan, 2016
Sialic acid and peptide-B6	Selenium nanoparticles	AD	Disaggregated the Aβ fibrils and inhibited the Aβ aggregation	Yin et al., 2015
Cysteine	Selenium nanoparticles	AD	↓ ROS, prevented Aβ aggregation	Zhou et al., 2015
Nattokinase enzyme (NK)	PNPs	AD	Downregulate amyloid aggregation	Lohan et al., 2017

## Green-Extract Nanoparticles

### Ginkgo biloba

*Ginkgo biloba* (Ginkgoaceae) is an ancient Chinese tree, extensively cultivated for traditional and medical purposes. *G. biloba* extract contains flavonol glycosides, bilobalide, terpene trilactones, and varied forms of ginkgolides, and ginkgolic acid (Müller et al., 2012; Yang et al., 2018). In Europe, the standardized form of *G. biloba* extract is broadly used to improve the therapeutic condition of patients with various forms of dementia (Maurer et al., 1997; Luo, 2001). It was reported that niosome formulation of *G. biloba* extract was able to extend the release duration of flavonoid glycosides with improved oral bioavailability and pharmacokinetic properties, making it an appropriate delivery system for *G. biloba* extract to the brain (Jin et al., 2013). Nanosized particles of *G. biloba* extract promoted the release of acetylcholine neurotransmitter from certain parts of the brain compared with control group animals. Nanosized particles of *G. biloba* extract showed improved bioavailability and a better absorption character (Shinji et al., 2011).

### Pomegranate Seed Oil

Pomegranate (*Punica granatum*) is a sacred fruit containing punicic acid (PA), and significant amounts of polyphenolic compounds (Kıralan et al., 2009; Vroegrijk et al., 2011; Boroushaki et al., 2016). In 2013, a nanodroplet formulation of pomegranate seed oil improved the Creutzfeldt Jacob disease (CJD). The results of the study exhibited that accumulation of scrapie isoform of the prion protein (PrPSc) did not show significant changes but neuronal loss and lipid oxidation relatively decreased, an indicative of neuroprotective function of pomegranate seed oil (Mizrahi et al., 2014). In mouse model of MS, nanodroplet formulation of pomegranate seed oil reduced the disease burden more than free pomegranate seed oil (Binyamin et al., 2015).

### Thymoquinone (TQ)

The major active component of *Nigella Sativa* (Ranunculaceae) seed is TQ. TQ a lipophilic compound with diverse pharmacological qualities in immunomodulation, neurodegeneration and cognitive deficits (Alam et al., 2012). Nonetheless, the brain delivery of TQ is a challenge (Xiao et al., 2016). In high-fat cholesterol diet rats, nanoemulsion of TQ rich fraction (TQRF) and TQ improved memory deficits and enhanced the total antioxidant status, whereas significantly decreased the Aβ expression (Ismail et al., 2017b). In a similar condition, TQRF nanoemulsion and TQ nanoemulsion modulated the activity of γ- and β-secretase enzymes, which consequently increasd the Aβ degradation and its elimination from the brain (Ismail et al., 2017a). Co-encapsulation of *N. sativa* oil (NSO) and plasmid DNA demonstrated that NSO could be used as a suitable gene delivery carrier for NDs treatment, especially in AD subjects (Doolaanea et al., 2016). In a study, TQ encapsulated chitosan NPs were tested for the nose to brain targeting method. Nose to brain targeting is a way to reduce the systemic adverse effect of TQ. The outcomes of the study confirmed the effectiveness of TQ, comparing with previous methods (Alam et al., 2012).

## Conclusion

In parallel with global improvement of lifespan, the prevalence of NDs is rising up, thereby, requiring novel treatment strategies to improve both the symptomatic and the quality of life in patients suffering from such disease. As known, the CNS is tightly preserved with various barriers. Thus, a proper drug essentially has to pass the BBB to reach the CNS. Nevertheless, numerous drug delivery systems designed and developed, however, phytochemical-based nanocarriers have distinguished advantages such as being safe, ecofriendly, less toxic, inexpensive, easy to scale up, and providing particles with controlled size and morphology. In sum, plant mediated nano systems can improve the pharmacokinetic profile and bioavailability of phyto-therapeutic compounds to the CNS, increase the brain penetration of these drugs, and enhance the disaggregation or prevent the aggregates formation in the brain. Although, there are many studies reporting the restorative effect of NPs in preclinical models of neurological disorders, further research is requisite to address the safety issues related to these systems. In addition, clinical efficacy of NPs in the area of neurological medicine needs long term assessments. Design of nanoformulations with more specificity for different brain cells and for each type of NDs should also be noticed.

## Author Contributions

SZM, MF, and MA designed the structure of the manuscript and drafted the manuscript. SM, MF, SZM performed the literature search and contributed in writing the manuscript. SZM, SM, and ZB, reviewed and revised the manuscript. All authors had full access to the final version of the manuscript and gave their approval before publishing.

## Conflict of Interest

The authors declare that the research was conducted in the absence of any commercial or financial relationships that could be construed as a potential conflict of interest.
